# Short-Term Molecular Acclimation Processes of Legume Nodules to Increased External Oxygen Concentration

**DOI:** 10.3389/fpls.2015.01133

**Published:** 2016-01-06

**Authors:** Ulrike Avenhaus, Ricardo A. Cabeza, Rebecca Liese, Annika Lingner, Klaus Dittert, Gabriela Salinas-Riester, Claudia Pommerenke, Joachim Schulze

**Affiliations:** ^1^Department of Crop Science, Section for Plant Nutrition and Crop Physiology, Faculty of Agriculture, University of GoettingenGoettingen, Germany; ^2^Departamento de Ingeniería y Suelos, Facultad de Ciencias Agronómicas, Universidad de ChileLa Pintana, Chile; ^3^Department of Developmental Biochemistry, DNA Microarray and Deep-Sequencing Facility, Faculty of Medicine, University of GoettingenGoettingen, Germany

**Keywords:** H_2_ evolution, nicotianamine synthase, nitrogenase, nitrogen fixation, nodules, RNAseq, oxygen, Tnt1-mutant

## Abstract

Nitrogenase is an oxygen labile enzyme. Microaerobic conditions within the infected zone of nodules are maintained primarily by an oxygen diffusion barrier (ODB) located in the nodule cortex. Flexibility of the ODB is important for the acclimation processes of nodules in response to changes in external oxygen concentration. The hypothesis of the present study was that there are additional molecular mechanisms involved. Nodule activity of *Medicago truncatula* plants were continuously monitored during a change from 21 to 25 or 30% oxygen around root nodules by measuring nodule H_2_ evolution. Within about 2 min of the increase in oxygen concentration, a steep decline in nitrogenase activity occurred. A quick recovery commenced about 8 min later. A qPCR-based analysis of the expression of genes for nitrogenase components showed a tendency toward upregulation during the recovery. The recovery resulted in a new constant activity after about 30 min, corresponding to approximately 90% of the pre-treatment level. An RNAseq-based comparative transcriptome profiling of nodules at that point in time revealed that genes for nodule-specific cysteine-rich (NCR) peptides, defensins, leghaemoglobin and chalcone and stilbene synthase were significantly upregulated when considered as a gene family. A gene for a nicotianamine synthase-like protein (Medtr1g084050) showed a strong increase in count number. The gene appears to be of importance for nodule functioning, as evidenced by its consistently high expression in nodules and a strong reaction to various environmental cues that influence nodule activity. A Tnt1-mutant that carries an insert in the coding sequence (cds) of that gene showed reduced nitrogen fixation and less efficient acclimation to an increased external oxygen concentration. It was concluded that sudden increases in oxygen concentration around nodules destroy nitrogenase, which is quickly counteracted by an increased neoformation of the enzyme. This reaction might be induced by increased formation of NCR peptides and necessitates an efficient iron supply to the bacteroid, which is probably mediated by nicotianamine.

The paper is dedicated to the 85th birthday of Prof. Dr. Günther Schilling, University of Halle/Wittenberg, Germany, https://de.wikipedia.org/wiki/Günther_Schilling

## Introduction

Legumes are of worldwide importance in agriculture. In comparison to other crops, legumes are less dependent on available soil mineral N and have a reduced need for N fertilization since symbiotic bacteria provide ammonium in exchange for a suitable environment in nodules with low internal oxygen concentration and ample nutrient and energy supply. Under soil conditions the O_2_ concentration around nodules is likely to vary due to respiration processes of soil organisms and changes in the soil water content. While a decreased O_2_ concentration might impede nitrogen fixation *via* a limited O_2_ supply for ATP production, an increased O_2_ concentration in the soil air might enhance the risk of nitrogenase destruction. Nitrogenase is a phylogenetically ancient enzyme that evolved under conditions of a reducing atmosphere (Boyd and Peters, 2013). Consequently, the enzyme is sensitive to oxygen since both components – the small Fe-protein (dinitrogenase reductase) and the larger MoFe-protein (dinitrogenase) - are irreversibly inactivated and destroyed by oxygen (Eady and Postgate, 1974; Dixon and Kahn, 2004). Furthermore, the expression cascade of genes for nitrogenase is initiated only at concentrations of oxygen in the low nM range (Ditta et al., 1987; Soupene et al., 1995; Sciotti et al., 2003). For this reason, a prerequisite for active nodules is that several measures, in particular an oxygen diffusion barrier (ODB) at the inner cortex of the nodule in conjunction with high nodule respiration (about three to four times the respiration of an equal amount of root biomass, Ryle et al., 1979), guarantee microaerobic conditions around nitrogenase (Layzell and Hunt, 1990). The O_2_ concentration inside the fixation zone of the nodule is maintained at about 18 nmol (Layzell et al., 1990), in contrast to an O_2_ concentration around the nodule of about 250 μmol (Thumfort et al., 1994).

Quick adjustments in nodule activity to rapid changes in oxygen concentration (Hunt et al., 1987, 1989; Delcastillo et al., 1992) and the fact that nodule permeability is altered under various stress conditions (Denison, 1998) have led to the concept of a highly elastic, quickly variable ODB that maintains a nearly constant oxygen concentration within the infected zone and can reduce or even shut down nodule activity under stress conditions by reducing the oxygen concentration in the nodule interior through reduced nodule oxygen permeability. Consequently, oxygen supply to the nodule interior is a principle factor in the regulation of nodule activity (Schulze, 2004).

The reduction of N_2_ by nitrogenase is necessarily accompanied by the reduction of H^+^. The resulting gaseous H_2_ evolves from the nodules and provides a continuous non-invasive measure of nitrogenase activity. Using this approach, detailed studies on the short-term reaction to increased external oxygen concentration of soybean (Hunt et al., 1989), pea and lupin nodules (Delcastillo et al., 1992) have been performed. A sudden increase in oxygen concentration around nodules consistently results in an immediate steep decline in nodule activity followed by a recovery to almost pre-treatment levels within about 30 min. The mechanism of the quick recovery was to date only attributed to a quick adjustment of the nodule oxygen permeability by a highly flexible ODB and a recovery of nitrogenase from a transient inhibition. Hunt et al. (1989) argue that, given the timeframe, a *de novo* synthesis of nitrogenase appears to be less likely than a recovery from a transient inhibition. The hypothesis of the present study was that, in addition to the activity of the variable ODB, the short-term reactions of legume nodules to shifting oxygen pressure involve further complex molecular acclimation processes, in particular those that induce increases in the *de novo* synthesis of nitrogenase.

Such quick molecular reactions have been described for other physiological processes. For instance, in *Arabidopsis* more than 500 genes are differentially expressed in roots 20 min after exposure to nitrate (Krouk et al., 2010). A rapid molecular reaction might be indicated in particular for bacteroids where nitrogenase activity constitutes the basic function and metabolic activity in the symbiotic stage. Furthermore, strong morphological reactions of legume nodules are reported under long-term altered oxygen pressure, which makes it appear unlikely that the variable ODB simply fully maintains unchanged oxygen concentrations within the nodules (Dakora and Atkins, 1991). Bergersen (1997) states that the concept of a variable ODB can only be a simplification because there are several metabolic components involved in the interacting regulation of N_2_ fixation and O_2_ consumption.

The aim of the experiments performed was in a first step to establish the time course of nitrogenase activity (H_2_ evolution) after an increase in oxygen concentration around nodules for the model legume *M. truncatula*. Subsequently, comparative transcriptome profiling (RNAseq) was performed on nodules after exposure to elevated oxygen at the point in time when the activity of the nodules was fully restored. This transcriptome profile was used as a screen for the study and for further functional characterisation of genes possibly involved in short-term acclimation of the nodules to elevated oxygen.

## Materials and Methods

### Design of the Experiments

The objective of the study was to test the hypothesis that molecular processes are involved in the acclimation of *M. truncatula* nodules to elevated external oxygen concentrations. Firstly the time course of nodule activity (H_2_ evolution) was followed with high time resolution before and during the exposure of the nodules to elevated external oxygen concentrations. Secondly a comprehensive comparative transcriptome profile of nodules was performed through next-generation deep sequencing (RNAseq). The nodules for the transcriptome profile were harvested at the point in time when, according to the first experiment, nodule H_2_ evolution had reached a new constant level under elevated external oxygen concentrations. In addition, nodules were harvested at key moments of the acclimation process to follow the expression of bacterial genes central for nitrogenase formation by qPCR. Lastly, the acclimation to elevated external oxygen concentration of a mutant that carries an insert in a key gene of nitrogen fixation was analyzed.

### Plant Growth

*M. truncatula* lines A17 and R108 were used for the experiments. The plants were grown with their roots and nodules in glass tubes with nutrient solution supplied from a large container in a circling flow. The procedure for seed preparation, early growth, subsequent growth in the glass tubes and the conditions in the growth chamber are described in Cabeza et al. (2015). The plants in the experiments showed vigorous growth. Apart from a low starter ammonium supply (one-time adjustment to 0.25 mM ammonium of 20 L nutrient solution for six plants), they depended solely on nitrogen fixation for N nutrition. The plants were inoculated with *Ensifer meliloti* (*Sinorhizobium meliloti*) 102F51 according to the procedure described in Cabeza et al. (2015). Earlier experiments have shown that additional application of the inoculum does not increase the nodule number. The inoculation resulted in healthy-looking pinkish nodules in sufficient numbers and high H_2_ evolution rates of the plants in all experiments. The *E. meliloti* strain has no genes for a hydrogen-uptake hydrogenase (hup^-^) (Blumenthal et al., 1997). The experiments were performed on 6 to 8-week old plants. By that point they had formed about 10 g dry matter per plant. The nodule number in the experiments was not counted. In experiments with according growth conditions and plant age, the nodule number was usually around 400–800 per plant.

### Measurement of Nitrogen Fixation

The plants with their roots in the glass tubes were embedded in a gas exchange measurement system. For that purpose the glass tubes were constantly aerated with a flow of 200 ml min^-1^ (0.8 volume min^-1^, including the nutrient solution-phase in the tubes) that did not mix with outside air before the measurement of H_2_ concentration. The set-up of the glass tubes is described in Fischinger and Schulze (2010) and the whole measurement system is described in Cabeza et al. (2015). The measurement system allowed the continuous non-invasive measurement of nodule H_2_ evolution at a high time resolution. This was achieved by measuring H_2_ concentration in a constant and defined flow through the root/nodule compartment that takes up H_2_ evolving from the nodules and provides a measure of nitrogenase activity. In these experiments the H_2_ concentration in the gas flow was measured every 12 s. In addition, the composition of the gas flow through the root/nodule compartment could be altered quickly and in all intended proportions. In the experiments the normal conditions N_2_/O_2_ (79/21, v/v) were altered to a concentration of 25 or 30% oxygen at the expense of nitrogen. The change took a few seconds and the airstream of the new composition reached the root/nodule compartment 2 min later. Nitrogen fixation as unit nitrogen was calculated from the apparent nitrogenase activity (ANA = H_2_-evolution in N_2_/O_2_, 79/21, v/v) and total nitrogenase activity (TNA = peak H_2_ evolution during 5 min exposure of nodules to Ar/O_2_, 79/21, v/v) as (TNA-ANA)/3 (Fischinger and Schulze, 2010). On the assumption that TNA reflects the total electron flow through the enzyme, the difference between TNA and ANA corresponded to the amount of electrons flowing onto nitrogen. The denominator of three is valid since H_2_ was being measured and the reduction of N_2_ to NH_3_ requires six electrons, while that of H^+^ to H_2_ requires one electron pair. The procedure for measuring N_2_ reduction has been validated through a parallel measurement of ^15^N_2_ uptake (Fischinger and Schulze, 2010).

### Nodule Transcriptome Profiling Through RNAseq

The first experiment showed that 30 min after the nodules started to be exposed to an elevated external oxygen concentration a new more or less constant level of activity was established. Consequently, at that point in time, molecular acclimation mechanisms should be active. A transcriptome profile was therefore performed on nodules exposed for 30 min to 25% oxygen *versus* those grown continuously in air with a 21% oxygen concentration. For this, 25 nodules per plant from nine plants in the treatment and the control were harvested. The nodules were collected using sterile forceps and immediately submerged in TRIzol (Invitrogen). The nodule collection took about 5 min per plant, during which time the nodules in the 25% treatment were kept in an atmosphere with an elevated oxygen concentration. The nodules were kept in the TRIzol at room temperature for 10 min in order to ensure that the TRIzol had fully penetrated the tissue. Subsequently the nodules in TRIzol were frozen at -80°C and kept at this temperature until RNA extraction (1 day). This procedure guaranteed the extraction of clean and undegraded RNA. After quality control, the RNA of nodules of three randomly chosen plants within treatment or control was combined. This resulted in three biological replicates in treatment and control. RNA extraction including quality control, the construction of a library based on poly-T-primer, sequencing, alignment of the read sequences, and the statistical treatment of read and count numbers, including the comparison of treatment and control, are described in Cabeza et al. (2014a). It should be noted that only ‘unique hits’ were counted in the alignment process. Furthermore, in contrast to the procedure in Cabeza et al. (2014a), the newly released gene model Mt4.0v1 was used for alignment instead of Mt3.5v3.

### qPCR

Total RNA was isolated using the TRIzol reagent (Invitrogen) as described by Hummon et al. (2007) (TRIzol/chloroform method). The integrity of the RNA was tested by gel electrophoresis. After DNase I (Invitrogen) treatment, the complementary DNA (cDNA) was constructed with random hexamer primers using the SuperScript^®^ III First-Strand Synthesis SuperMix (Invitrogen, Life Technologies, Thermo Fisher Scientific, USA) with the StepOne thermo cycler (Applied Bioscience, Life Technologies, Thermo Fisher Scientific, USA). Subsequently the Fast SYBR^®^ Green Master Mix (Applied Biosystems, Life Technologies, Thermo Fisher Scientific, USA) and the StepOne thermo cycler were used for comparative real-time PCRs. The PCR reaction mix was prepared in a 48-well plate according to the manufacturer’s instructions. All templates were amplified using the following PCR protocol: 95°C for 20 s; 40 cycles of 95°C for 3 s and 60°C for 30 s, and SYBR Green fluorescence was measured continuously. The measurement was concluded with a melting curve: 95°C for 15 s, 60°C for 1 min, slow temperature increase for 34 min up to 95°C, and 95°C for 15 s. The following bacterial genes were tested: *nifA*, *nifK*, *fixK* and *nifE*. The experiment was performed with five biological treatments, which were each measured twice. The primers (Invitrogen, Life Technologies, Thermo Fisher Scientific, USA) are shown in **Supplementary Table S1**. SMc00128 (Glenn et al., 2007) and SMc02676 (Viguier et al., 2005) were used as housekeeping genes. The primers were designed using the NCBI Primer-BLAST^1^.

### Statistical Analysis of Differential Expression of Whole Gene Families

The expression of all individual genes annotated in the gene model Mt4.0v1 was compared between treatment and control using the DESeq method. The Mann–Whitney *U* test was applied at the 0.05 level of significance to evaluate differences of a gene family as a whole. The Mann-Whitney *U* test is recommended for comparing the expression of a group of genes of the same family under two conditions (De La Torre et al., 2015). Statistical analyses were performed using Statistica 10.0 (StatSoft, Inc. Tulsa, OK, USA).

## Results

### Short-Term Reaction of Nodule H_2_ Evolution to Increases in Oxygen Concentration

**FIGURE 1:**
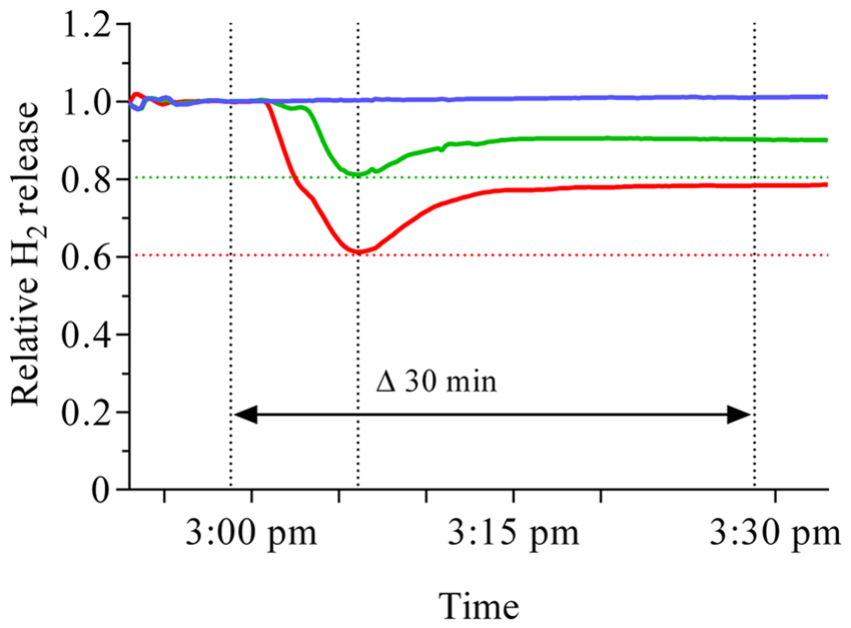
**Time course of nodule H_2_ evolution before and after an increase in external oxygen concentration around *M. truncatula* nodules.** Data were taken every 12 s for a time period of about 45 min, including 30 min after the increase in external oxygen concentration. The blue line represents the untreated control, while the external oxygen concentration represented by the green or red line was increased from 21% to 25 or 30% respectively. The increase was implemented at 2:58 pm, indicated by the dotted line. A steep decline started about 4 min after increasing the external oxygen concentration and leveled off after 10 min (second dotted line from the left), irrespective of the extent of the increase in oxygen concentration. A subsequent recovery reached a more or less constant new level about 15 min after the change in external oxygen concentration. The extent of the initial decline is indicated by the red and green dotted lines.

A sudden increase in external oxygen concentration resulted in a repeatable pattern of the reaction of nodule H_2_ evolution in *M. truncatula* which was consistent with reports about this reaction in soybean, pea and lupin (Hunt et al., 1989; Delcastillo et al., 1992). About 2 min after the nodules were exposed to either 25 or 30% oxygen, a steep decline in activity occurred (**Figure 1**). The relative extent of the decline in nodule activity was moderately proportional to the change in O_2_ concentration. However, irrespective of the size of the increase or the initial oxygen concentration, about 10 min after exposure to the elevated oxygen concentration, the decline ceased and an increase in H_2_ was observed. A further 5 min later the increase began to ease off and led to a more or less constant level. Within the studied time of about 30 min, the activity had not fully recovered to its pre-treatment level.

### Short-Term Increase in the Expression of Genes for Nitrogenase

**FIGURE 2:**
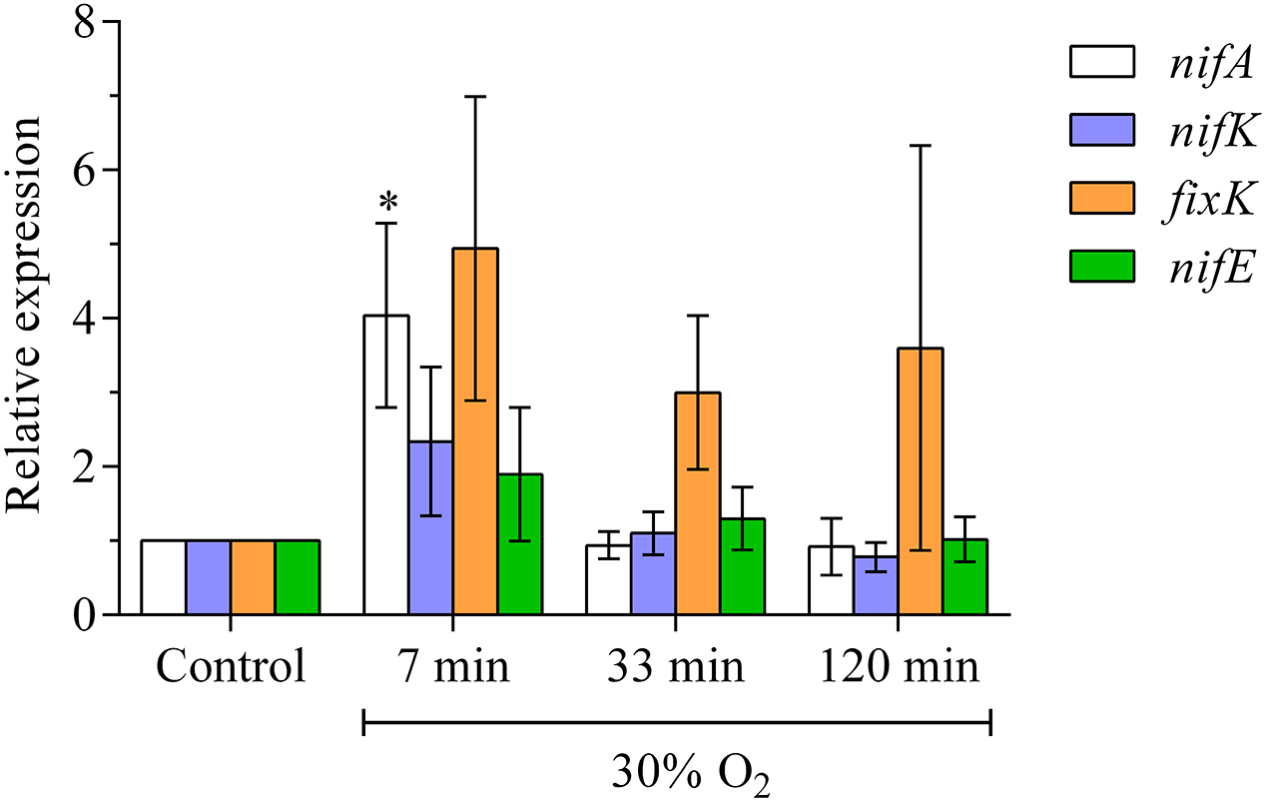
**Expression of bacterial genes for structural and regulatory components of nitrogenase formation during exposure of nodules to elevated external oxygen concentrations.** Relative transcript abundance of bacterial genes in nodules treated with 30% O_2_ for 7, 33, or 120 min. Each column is the average of five biological replicates. Ct values were normalized against two housekeeping genes (SMc00128 and SMc02676). Error bars show standard errors. ^∗^ means a statistical significant difference when compared to the control (two-way ANOVA, *n* = 5, *p* < 0.05).

*NifA*, *nifK*, *fixK* and *nifE* showed a rapid increase in expression at the point in time, when the H_2_ evolution of the nodules starts to recovers (**Figure 2**). While the expression of *nifA*, *nifK*, and *nifE* returned to pre-treatment levels at 30 min after exposure to elevated oxygen*, fixK* remained more strongly expressed, also after 120 min of exposure to elevated oxygen.

### Nodule Transcriptome Profile After 30 min’ Exposure to 25% External Oxygen Concentration

**FIGURE 3:**
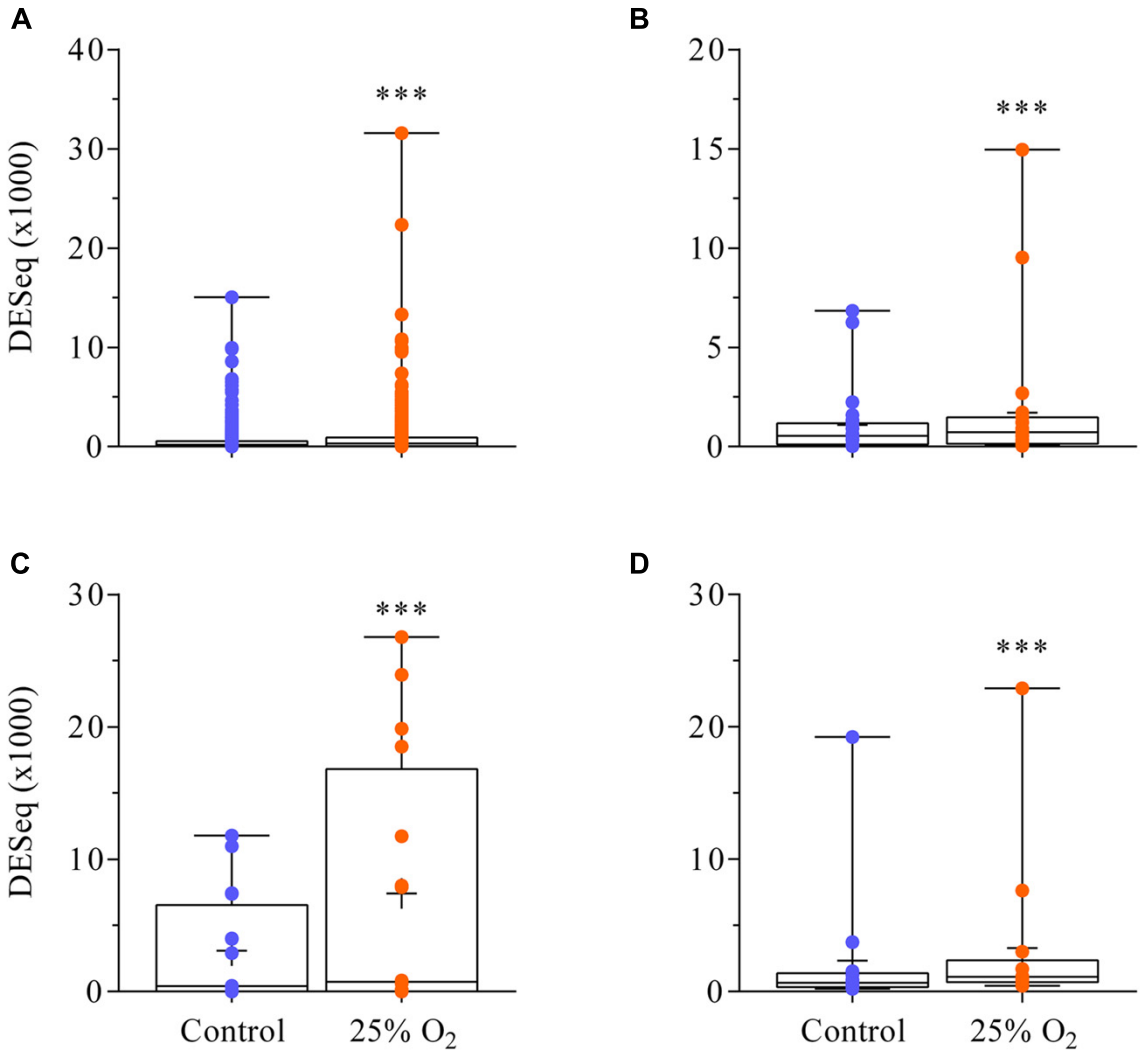
**Upregulation of the gene families for NCR peptides, defensins, leghaemoglobins and chalcone and stilbene synthase 30 min after an increase in external oxygen concentration around *M. truncatula* nodules.** 499 transcript units were found for NCR peptides **(A)**, 29 transcript units for defensins and defensin-related peptides **(B)**, 11 transcript units for leghaemoglobin **(C)**, and 13 transcript units for chalcone and stilbene synthase **(D)** expressed in the nodules (<20 ‘unique hit’ DESeq counts in either the control, the treatment or both). Although none of these transcript units was individually significantly increased in abundance **(Supplementary Table S2)** each gene family was upregulated. ^∗∗∗^ means a statistically significant difference when compared to the control (Wilcoxon test, *n* = 3, *p* < 0.001).

The RNA sequencing yielded about 10–15 million reads per replicate and thus achieved the necessary depth for this analysis. A total of 16 615 transcript units were found in an abundance that resulted in >20 DESeq ‘unique hit’ counts in the control, the treatment or both (**Supplementary Table S2**). According to that threshold, 26.7% of all transcript units annotated in the gene model Mt4.0v1 were expressed in nodules. Genes yielding lower DESeq count numbers for their transcript units were considered silent. No individual transcript unit was significantly altered in transcript abundance (FDR ≤ 0.01) by the impact of 30 min of elevated external oxygen concentration (21 versus 25% oxygen). However, 69 transcript units showed a log_2_ fold change greater than [2] (**Supplementary Table S3**). This group of genes could be used as a starting point for further studies into their possible role in acclimation to elevated external oxygen. When gene families were considered, a significant upregulation of genes for NCR peptides, defensins, leghaemoglobins and chalcone and stilbene synthase family proteins could be observed (**Figure 3**). **Supplementary Table S4** contains the normalized counts of the three replicates in control and treatment for all transcript units annotated in the gene model Mt4.0v1.

### A Mutant Carrying an Insert in the Coding Sequence of a Nicotianamine Synthase-Like Protein (Medtr1g084050) Showed Impaired Nitrogen Fixation and Less Efficient Acclimation to Changing External Oxygen Pressure

#### Nitrogen Fixation

**FIGURE 4:**
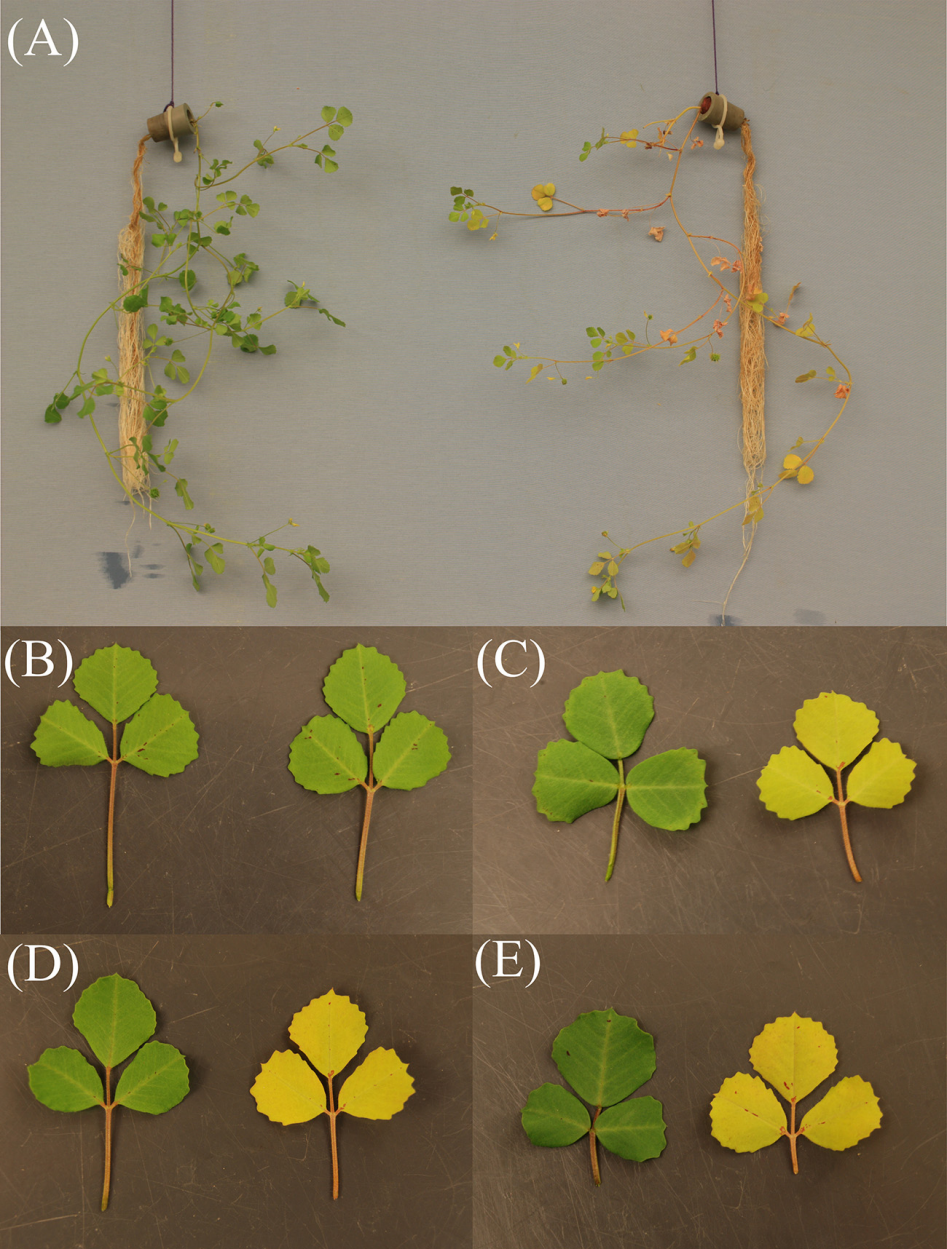
**Growth of a Tnt1-*Mt*-mutant carrying an insert in the cds of Medtr1g084050 (nicotianamine synthase-like protein).** Three-week old nodulated plants of the mutant were transferred to glass tubes with N-free nutrient solution. The plants received a low (0.25 mM) ammonium start-N supply during the first week of growth in the tubes and relied exclusively on N_2_ fixation for the next 7 days. During the subsequent 2 weeks, for one set of plants the nutrient solution was adjusted once a week to 5 mM nitrate concentration, while the other plants relied solely on nitrogen fixation. **(A)** Shows nitrate (left) and N_2_-supplied (right) plants at the end of the growth period. **(B–E)** Show fully expanded leaves of nitrogen fixation (right) or nitrate-dependent (left) plants 0, 7, 10, and 14 days after the beginning of the nitrate supply.

**FIGURE 5:**
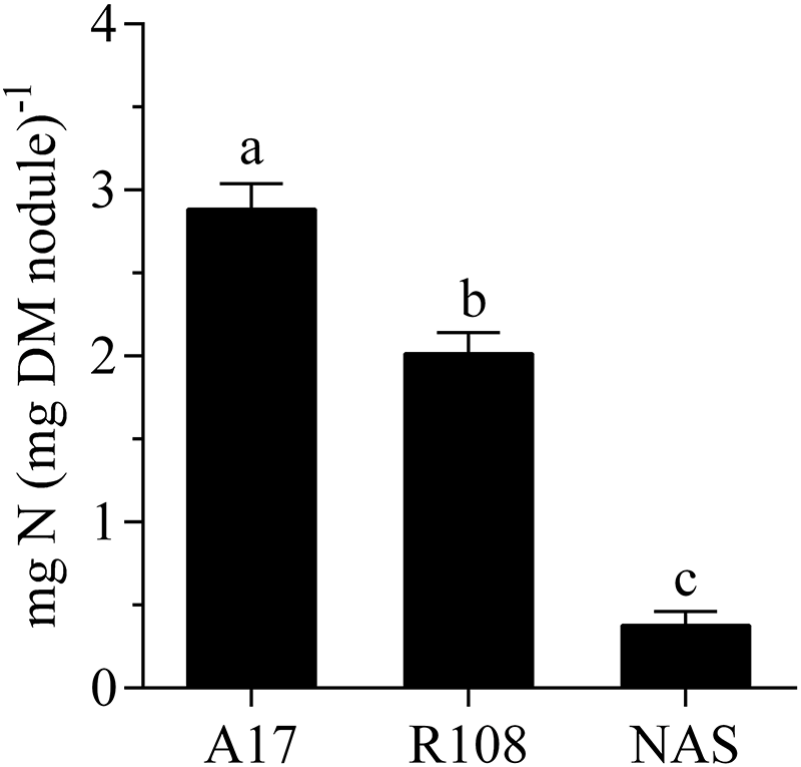
**Specific nitrogen fixation of nodules of A 17 and R108 wild type plants and a Tnt1-*Mt*-mutant carrying an insert in the cds of Medtr1g084050 (nicotianamine synthase-like protein).** The data were obtained from a continuous measurement of nodule H_2_ evolution (ANA) over a 24 h period and a point measurement of H_2_ evolution in an Ar/O_2_ mixture (79/21, v/v) at 10 am. Data are means of six independent biological replicates. Error bars represent standard errors. Different letters indicate a significant difference (Tukey’s test; *p* < 0.05).

The gene Medtr1g084050, annotated as nicotianamine synthase-like protein in the gene model Mt4.0v1, showed a strongly increased count number after the nodules had been exposed to an elevated external oxygen concentration for 30 min. This occurred from the second highest mean expression level among the transcript units with a fold change in abundance greater than log_2_ [2] (**Supplementary Table S3**). Given the great importance of iron supply for nitrogen fixation and the strong reaction of the gene in a prior study on the effect of nitrate on nodules (Cabeza et al., 2014a), this particular gene was chosen for more detailed studies. Nitrogen fixation and acclimation of nodules to changing external oxygen concentration of a Tnt1-mutant that carries an insert in Medtr1g084050 (FST:NF 11218^2^) were studied. The mutant developed normal-looking, pinkish nodules in numbers comparable to those on plants of the wild type under the conditions of this study’s growth system. However, when depending solely on nitrogen fixation for N nutrition, the plants developed N-deficiency symptoms (**Figure 4**). The fact that these symptoms could be rescued by nitrate application showed that the mutation had an impact on nitrogen fixation rather than on the supply of iron to the leaves. This was further supported by measurements of the specific nitrogen fixation rates, which revealed that the specific activity of the mutants’ nodules was several times lower than in the wild type or nodules of the model line A 17 (**Figure 5**).

#### Reaction of the Mutant’s Nodules to Changing External Oxygen Concentrations

**FIGURE 6:**
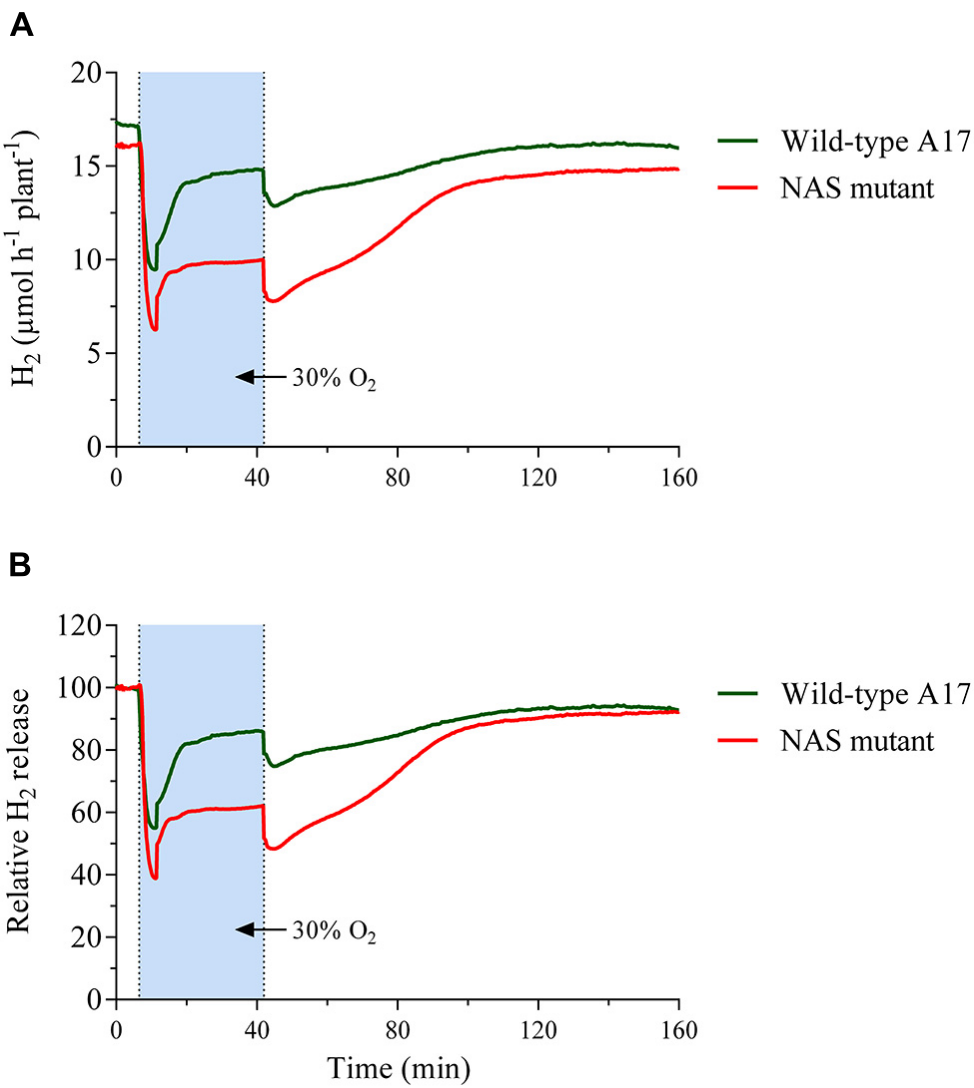
**Time course of nodule H_2_ evolution during changes in the external oxygen concentration around nodules of *Mt*-wild type and mutant plants, carrying an insert in the cds of Medtr1g084050 (nicotianamine synthase-like protein).** Data were taken every 12 s for a period of about 160 min, including 30 min of elevated external oxygen concentration. **(A)** Shows nodule H_2_ evolution as μmol H_2_ per hour and plant, while the data in **(B)** are given as % of the value at the beginning of the shown measurement. Data are the mean value of five replicates. The plants of the wild type and the mutant were five and 10 weeks old respectively, at which age they had comparable per plant nitrogen fixation rates.

The fundamental pattern of nodule H_2_ evolution during an increase in external oxygen concentration resembled that of the wild type in that the initial decline turned into an increase about 9 min after exposure and a new relatively constant level was reached after about five to 10 min later (**Figure 6A**). This was measured from a comparable per plant nitrogen fixation level. The mutant plants required about double the growth period to reach that level. The mutants’ growth was characterized through slow and gradually increasing formation of new leaves, supported by a comparatively low input of N from the nodules and additionally N remobilised from old leaves. Accordingly, contrary to the wild type, old leaves of the mutant quickly senesced and were eventually shed off. When the timeframe of H_2_ evolution was expressed as a percentage of the activity before the increase in external oxygen concentration, it became apparent that the initial decline was about one third greater and the subsequent recovery reached a much lower level (**Figure 6B**). However, when the external oxygen concentration was restored to ambient concentration, the mutant’s nodules showed a steeper increase and recovery to activity levels similar to those before the treatment in a timeframe comparable to the wild type.

## Discussion

### An Extended Model for the Mechanisms of Nodule Acclimation to Elevated External Oxygen

**FIGURE 7:**
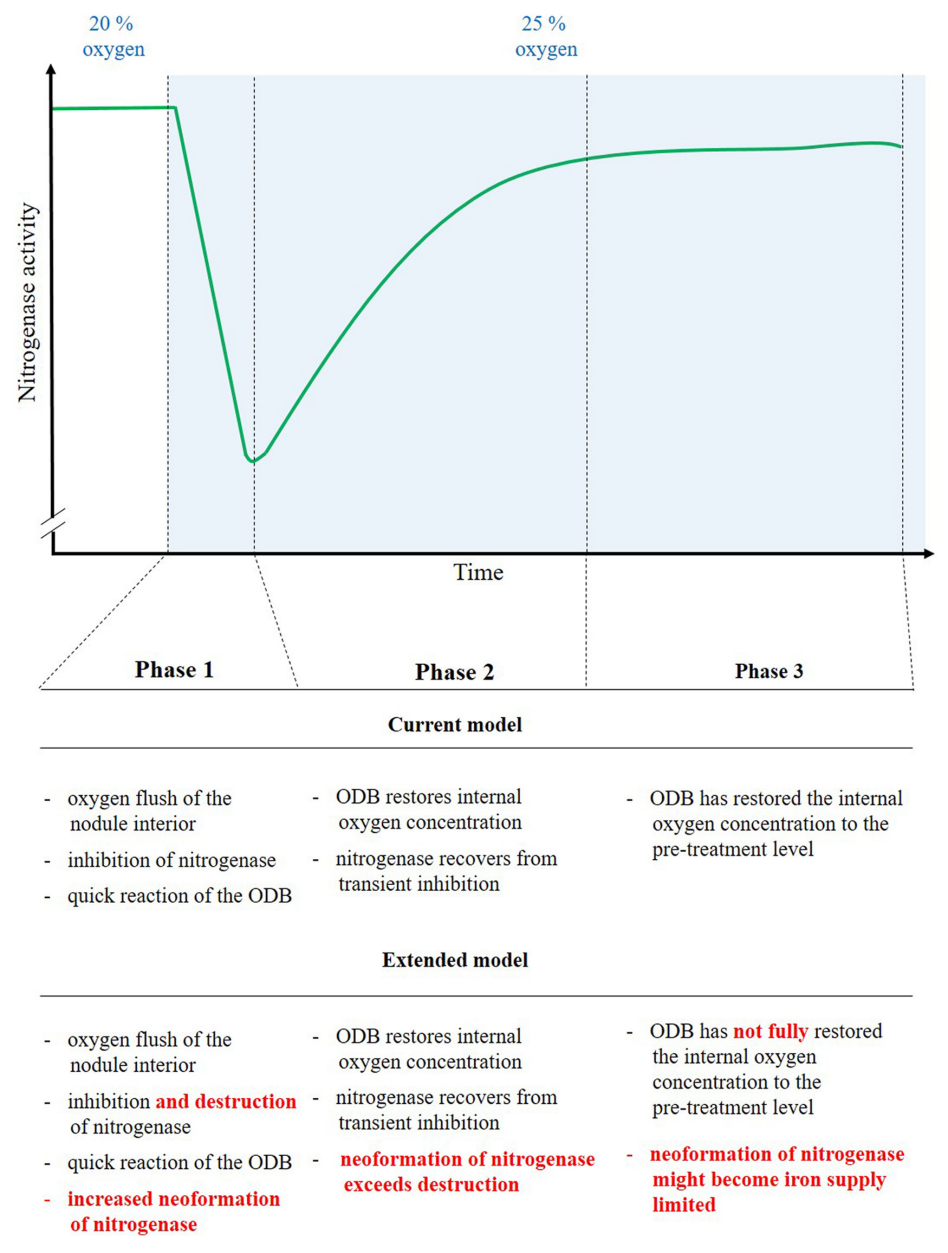
**Reasons for the time-course of nitrogenase activity (H_2_ evolution) before and after an increase in external oxygen concentration.** The green line stands for nitrogenase activity (H_2_ evolution). The time-period after the increase in external oxygen concentration is blue-shaded. The time-period is divided in three phases with a distinct course of nodule activity. Phase 1, as the immediate time after the increase in oxygen concentration, is characterized by a quickly starting (after about two 2 min) steep drop in H_2_ evolution. During phase 2 the nodule activity recovers quickly, while during phase 3 a new, more or less constant nodule activity is reached. This new level of activity is slightly below the pre-treatment level. For each individual phase, the explanation for the behavior of nodule activity in the current model and the extension of that understanding based on our data are given. The extensions are highlighted in red color.

Our data suggest that increases in external nodule oxygen concentration are connected to an oxygen flush of the nodule interior and a resulting destruction of nitrogenase. As a consequence the steady state of nitrogenase turnover is disrupted, resulting in a steep decline in nodule H_2_ evolution (**Figure 7**, phase 1). Part of the reason for this effect might also be a transient inhibition of a fraction of intact nitrogenase. Within a timeframe of 10 min a strong counter-reaction on a transcriptional level occurred through an upregulation of genes for nitrogenase formation. The reaction might be induced by an increased allocation of NCR peptides to the bacteroids. The increased *de novo* synthesis of nitrogenase counterbalanced the oxygen-induced nitrogenase destruction. The process was supported by a tightening of the ODB, with the resulting decreasing internal oxygen concentration, less nitrogenase destruction and a possible release of transiently inhibited nitrogenase protein. Together these factors turned the steep initial decline into an increase as early as 10 min after the switch to an elevated external oxygen level (**Figure 7**, phase 2). The increase reached a new more or less constant level some 15–20 min after the switch to a higher external oxygen concentration (**Figure 7**, phase 3). At this point in time the internal oxygen concentration, although downregulated by the ODB, still appeared to be higher than under pre-treatment conditions. This is supported in particular by increased transcript abundance of leghaemoglobin, also by the fact that nodules show long-term adaptations to elevated external oxygen concentrations. The higher internal oxygen concentration still led to increased nitrogenase destruction and the new steady state is characterized by a higher nitrogenase turnover. The increased *de novo* nitrogenase synthesis needed an increased iron supply, which is highlighted by the increased expression of NAS in the wild type and a low level of the new steady state in the NAS mutant. At elevated oxygen concentrations, the new steady state level is apparently limited by the iron supply and allocation.

### Time Course Pattern of H_2_ Evolution of Nodules After Sudden Changes in External Oxygen Concentration

Legume nodules have been reported to acclimate quickly to increasing external oxygen concentrations. A steep initial decline of nodule H_2_ evolution (nitrogenase activity) was followed by a recovery that almost fully restored the nitrogenase activity. The reason for the initial decline was an oxygen flush of the nodule interior that impacted nitrogenase. The oxygen flush of the nodule interior can be measured indirectly by monitoring the oxygenation status of leghaemoglobin in the infected zone (King et al., 1988; Denison et al., 1991). A subsequent rapid tightening of the ODB released nitrogenase from a transient inhibition. There are no alternative hypotheses for the oxygen flush impact on nitrogenase but a destruction or transient inhibition of the enzyme. However, while it is well documented that nitrogenase is irreversibly destroyed by exposure to continuously high oxygen concentrations (Dixon and Kahn, 2004), the mechanism of a postulated initial, transient inhibition of the enzyme remains speculative. Nevertheless, such a transient inhibition of nitrogenase and a quick restoration of the oxygen concentration before the treatment in the nodule interior through rapid tightening of a flexible ODB are the cornerstones for the current explanation for the nodules’ quick acclimation to increased oxygen pressure (**Figure 7**). A possible additional increased *de novo* nitrogenase formation as a reaction to nitrogenase destruction has so far not been studied, but was considered unlikely given the swift reaction of nodule H_2_ evolution (Hunt et al., 1989). A possible rapidly functioning mechanism for the ODB might be driven by potassium fluxes between the nodule interior and the cortex and concomitant water fluxes and/or shrinking and swelling of cell layers (Wei and Layzell, 2006). The high elasticity of the nodule ODB within minutes was further supported by the fact that nodule H_2_ evolution remains almost unaffected when an increase in oxygen concentration occurs slowly and continuously over a 30 min period (Hunt et al., 1989). A rapid reaction of the ODB is furthermore in line with the results of indirect measurements of the oxygen concentration in the nodule interior from continuous monitoring of the leghaemoglobin oxygenation status (Denison et al., 1991). Such measurements show that after an initial oxygen flush of the nodule interior, the oxygen concentration quickly declines (despite increased external levels being maintained). Nevertheless, the measurements were insufficiently precise to show that the interior oxygen concentration was indeed maintained at that exact low level before the increase in oxygen concentration around the nodules. It is unclear whether even small shifts in oxygen concentration in the nodule’s interior might impact nitrogenase and the overall nodule functioning.

The principle reaction pattern of nitrogenase activity (H_2_ evolution) of *M. truncatula* nodules during increases in oxygen concentration resembled that reported for soybean, pea and lupin (Hunt et al., 1989; Delcastillo et al., 1992). An initial steep decline was immediately followed by a steep recovery. This recovery resulted in an activity close to the level before the change in external oxygen concentration. Nevertheless, there were minor differences. The recovery in activity of *M. truncatula* nodules occurred more or less continuously, in contrast to a ‘wave-like’ pattern reported for soybeans. Furthermore, the activity did not fully reach pre-treatment levels within the studied 30 min timeframe.

### Increased *de novo* Synthesis of Nitrogenase was Part of the Reaction to Elevated External Oxygen Concentration and could be Induced Through Increased Formation of NCR Peptides

At the point at which nodule H_2_ evolution began to recover (about 10 min after the increase in oxygen concentration), the expression of various bacterial regulatory genes for nitrogenase expression and genes for nitrogenase components were upregulated. Bacterial gene expression is known to be capable of rapid reactions to shifting environmental conditions. This process apparently counterbalances the oxygen flush-induced destruction and inhibition of nitrogenase that results in the initial steep decline in nitrogenase activity. This appears to contradict the fact that the expression of genes for nitrogenase necessitates low oxygen concentration in the nodules’ infected zone (Soupene et al., 1995). According to our data, the increase in oxygen concentration after the switch to a 25% oxygen concentration affected the nitrogenase protein, but did not decrease nitrogenase expression. The expression was even increased 7 min after the increase in external oxygen concentration. One possible reason for this is that a quick acclimation of the ODB restored a sufficiently low oxygen concentration as early as 7 min after the increase in external oxygen concentration. In particular surprising was the strong reaction of *fixK*. The gene was more strongly expressed after 7 min exposure to elevated oxygen and remained in tendency so during the 2 h elevated external oxygen. This is an unusual finding since the gene is involved in the low-oxygen dependent gene expression cascade that induce *fixA* expression. Furthermore, *fixK* is involved in various reactions of the bacteria in response to stress (Fischer, 1994). The strong reaction of *fixK* suggests that the gene may have some yet unknown functions in *rhizobia*. Overall, according to this study’s data, low oxygen concentrations were particularly important for the integrity of the enzyme and might reduce nitrogenase expression, but only after exceeding a level of about 50% external oxygen. In this case, no recovery in nitrogenase activity was observed and the nodules gradually lost their activity.

The recovery of nodule activity reached a new more or less constant plateau after about 30 min. The plateau was close to 90% but did not fully reach the pre-treatment level in H_2_ evolution. A comprehensive nodule transcriptome profiling at that point in time (30 min after exposure to 25% oxygen) revealed a significant upregulation of a large family of nodule-specific genes for small NCR peptides. These peptides are related to defensins and play a crucial role in the higher-plant governed regulation of bacteroid activity (Wang et al., 2010; Wang and Dong, 2011). Twenty three genes annotated as defensin (5) or defensin-related (18) are concertedly upregulated, concomitantly with the NCR peptides. In the inverted repeat-lacking clade (IRLC) of legumes, NCR peptides are one tool of the higher plants to force (sanction or punish) (Oono et al., 2009; Oono et al., 2011) the bacteroids to produce nitrogenase in amounts vastly in excess of their own needs. In this way, the higher plant ensures that the huge energy expenditure for nodule formation and activity is repaid by a continuous ammonia/ammonium flux to the higher plant. According to the data from the present study, the expression of genes for NCR peptides in *M. truncatula* nodules reacted either to increased oxygen concentration or to the destruction of nitrogenase and the concomitant decrease in ammonia formation. The increased expression of NCR peptides and defensins might be the set-point for counteracting the increased destruction of nitrogenase through an immediate increase in the flow of NCR peptides to the bacteroids. These peptides induce an increased formation of *nif*- and *fix*-gene transcripts. The role of NCR peptides in nodules of *M. truncatula* as the set-point in the regulation of nodule activity (nitrogenase formation) was furthermore shown by the fact that these peptides were rapidly and strongly downregulated under the impact of nitrate, a treatment that resulted in a quick decrease in nodule activity (Cabeza et al., 2014a).

### The Variable Oxygen Diffusion Barrier Did Not Appear to Restore the Low Internal Oxygen Concentration of the Nodules Fully

Another concertedly upregulated gene family was that of leghaemoglobin genes. Leghaemoglobin is abundant in the infected zone of nodules and provides the inner active nodule with its deep red color. In *M. truncatula*, genes for leghaemoglobin are among the most strongly expressed genes in nodule tissue (Cabeza et al., 2014a,b). RNAi of a leghaemoglobin gene in *Lotus japonicus* results in white fix^-^ nodules that have lost the capacity to fix nitrogen (Ott et al., 2005). Eleven genes for leghaemoglobin are annotated in the gene model Mt4.0v1. All these genes were strongly expressed in the control, and the transcript abundance more than doubled (log_2_ FC +1.2) on average after 30 min exposure to increased external oxygen concentration. Leghaemoglobins have two main functions in nodules (Mylona et al., 1995). Firstly, they bind oxygen and facilitate transport to the high-affinity bacteroid cytochrome oxidases. This might be a prerequisite for the efficient functioning of these bacterial cytochromes and for high respiration rates under the microaerobic conditions of the nodule interior. Secondly, leghaemoglobins function as a buffer against high oxygen concentrations or at least as an efficient means of lowering the concentration of free oxygen in the nodule interior (Appleby, 1984). Consequently, the increased expression was understandable in the context of the early oxygen flush of the nodule interior after the increase in external oxygen concentration. However, the fact that this increased expression was found 30 min after the initial change in external oxygen concentration indicated that the variability of the ODB alone did not fully restore the internal pre-treatment oxygen concentration. This assumption was supported by the fact that nodules that had undergone long-term treatments with elevated external oxygen concentrations showed strong morphological reactions that might represent acclimation processes to continuously higher internal oxygen concentrations. These morphological adaptations would not be necessary if a variable ODB alone were able to maintain constant internal oxygen concentrations. The mechanism of the functioning of that variable reaction is not fully understood (Hunt and Layzell, 1993; Minchin, 1997). Microscopic studies, supported by the use of various gaseous dyes, suggest that there are interconnected air-filled passageways of intercellular spaces reaching the infected zone of the nodules (Bergersen and Goodchild, 1972; Jacobsen et al., 1998). However, measurements with oxygen microelectrodes convincingly show that a considerable oxygen diffusion resistance occurs over several cell layers of the nodule inner cortex, lowering the oxygen concentration from a mM range to a low nM range (Tjepkema and Yocum, 1974). Since gas diffusion through gas phases encounters much less resistance than diffusion through liquid phases, nodules could limit O_2_ influx *via* water release into the intercellular spaces (Hunt and Layzell, 1993). The water movement could be induced by osmotic substances, for example sucrose (Layzell and Hunt, 1990), nitrogenous metabolites (Sheehy and Webb, 1991) or inorganic ions (Sheehy et al., 1983). Wei and Layzell (2006) show specific fluxes of potassium (K^+^) from the infected zone of the nodule to cortex cells under various treatments that are known to decrease nodule oxygen permeability. In turn, a decreased oxygen concentration around the nodules results in K^+^-fluxes from the cortex to the central infected zone. These K^+^-fluxes might be accompanied by a water flow that alters the oxygen permeability of the cortex. Furthermore, a guard cell-like shrinking and swelling of cortex cells could function as a variable component of the diffusion barrier, possibly regulated by the extent of malate formation in the involved cell layers of the nodule’s cortex (Schulze et al., 2002).

In this study’s experiments, nodule activity recovered during the experiments, but did not fully reach pre-treatment levels. This was most pronounced with nodules of the Tnt1-mutant. Although these results remain indirect, taken together with the elevated expression of leghaemoglobins they strongly indicate that after 30 min exposure to elevated external oxygen concentrations, the low pre-treatment internal oxygen concentration was not fully restored.

### After the Initial Recovery During Exposure to Elevated Oxygen Concentration, Nodule Activity Became Limited by the Iron Supply

After a short (30 min) impact of increase in external oxygen concentration by about 20%, the transcriptome profile did not show any individual gene with significantly altered transcript abundance. Nevertheless, the data proved to be useful as a screen for the selection and further study of potentially affected individual genes. One possible approach would be to consider genes with a strong expression fold change in the experiment. When setting a log_2_ FC of >[2] as a threshold, a total of 69 transcript units showed stronger changes (**Supplementary Table S3**). Among them, Medtr1g084050, a gene annotated as nicotianamine synthase-like protein in the Mt4.0v1 gene model, was of interest for several reasons. The expression of that gene was increased by a log_2_ fold change of +2.8. This strong fold change occurred from the second highest mean expression among the 69 mentioned transcript units with a high fold change, but did not reach significance levels when setting FDR ≤0.01 as a threshold. A number of RNAseq-based transcriptome profiling studies were performed on *M. truncatula* nodules under the impact of various environmental factors. In these experiments, the gene Medtr1g084050 was consistently the by far most strongly expressed gene among the five genes annotated as nicotianamine synthase or nicotianamine synthase-like genes in the gene model Mt4.0v1. The gene was upregulated when the nodules are exposed to 25% oxygen for 5 days (own unpublished data). Furthermore, the gene was strongly downregulated after 4 h of nitrate impact on the nodules, a treatment that reduces nitrogenase activity (Cabeza et al., 2014a). This was particularly surprising as an increased expression of nicotianamine synthase in roots of *Arabidopsis thaliana* after the impact of nitrate has been observed (Wang et al., 2003). A downregulation of Medtr1g084050 in nodules also occurred when nodule activity declined, induced by a darkening of the shoots in the normal light period (own unpublished data). Nicotianamine chelates iron and other micronutrients, thereby facilitating inner plant and inner cell transport. The nicotianamine-iron complex furthermore prevents the formation of oxygen radicals through free iron. Cells in the infected zone need a considerable supply of iron for leghaemoglobin and ferredoxin, but in particular for nitrogenase formation (Brear et al., 2013). Iron is an important structural and functional component of both nitrogenase subunits and is involved in electron transfer through the enzyme. Sufficient iron supply is a prerequisite for intensive nitrogen fixation of legumes. This is highlighted by the fact that emerging iron deficiency in legumes is often indicated through N deficiency as a result of impaired nitrogen fixation. Seeds of legumes are particularly rich in iron and molybdenum in order to support the developing nodules of young plants (Gurley and Giddens, 1969). The fact that on the one hand free iron is harmful to cells, but, on the other hand, that the maintenance of the nitrogen fixation apparatus requires a high iron supply and turnover, means that nodule cells have to have an efficient iron transport system. Nicotianamine is an important component of such a system and nicotianamine synthase is a central enzyme in its biogenesis. For these reasons the nitrogen fixation of a *M. truncatula* mutant-line that carries a Tnt1-insert in the cds of Medtr1g084050 was studied. The mutant showed a clear phenotype in that nodulated plants in N-free nutrient solution culture developed strong N deficiency that could be rescued by nitrate supply (**Figure 4**). Although the nodules appeared normal and developed the typical pink color, the specific activity measured was clearly lower when compared to the wild type (**Figure 5**). In addition, the mutant line showed a markedly different reaction to a sudden exposure to an elevated external oxygen concentration (**Figure 6**). The overall pattern of the time course of H_2_ evolution during the first 30 min of exposure to elevated oxygen resembled that of the wild type, but the initial decline was much stronger and, in particular, the new more or less constant level after the recovery from the initial decline was clearly below the pre-treatment activity. However, when the oxygen concentration was returned to 21%, a recovery to pre-treatment levels occurred swiftly. These results indicated that the new constant level of activity under elevated external oxygen concentrations was characterized by increased nitrogenase turnover, concomitant with increased leghaemoglobin formation. The limiting factor for both processes – at least for nitrogenase formation – appeared to be the iron supply, indicated by a stronger expression of NAS in the wild type and lower nitrogenase activity in the mutant. When the oxygen pressure was relieved, the activity of the mutant recovered quickly to the pre-treatment level, but was still low when compared to the wild type.

Mutagenesis imposed through transposons is based on the fact that a number of transposons insert themselves into the host genome by chance, particularly in gene-rich regions. Consequently, a phenotype does not necessarily result from a detected disruption of one gene, but might be the result of the impact of other, possibly undetected inserts elsewhere in the genome. There are various ways of increasing the probability that a phenotype does indeed result from the disruption of the gene in question. One possibility would be to use an independent mutant line that also carries an insert in the cds of the gene in question. If this line develops a comparable phenotype, the probability of a dependence of the phenotype on the gene strongly increases. The same is true for the possibility of generating backcrosses that retain the insert and may or may not develop the phenotype. In this study’s case, the facts that the gene was strongly expressed in nodules (1), that the expression is strongly downregulated under treatments that reduce nitrogenase activity (e.g., nitrate, Cabeza et al., 2014a) (2), that the expression was affected by elevated external oxygen concentrations in a predictable fashion (increase due to increased destruction of nitrogenase) (3) and finally the highly specific and consistent reaction of the mutant to elevated external oxygen concentration (4) make it highly probable that the described phenotype did indeed result from the disruption of Medtr1g084050.

### Module-Like Shifts in the Expression of Gene Families

The impact of 30 min of elevated external oxygen concentrations had significant effects on the expression of gene families. We have discussed the increased transcript abundance of genes for NCR peptides/defensins and leghaemoglobins. These reactions were consistent with the physiological and molecular results of the experiments. In addition, a further significant increase in the expression of genes annotated as ‘chalcone and stilbene synthase family proteins’ was observed. A total of 13 of such transcript units were almost doubled in abundance (log_2_ FC +0.8). Chalcone synthase catalyzes the first, possibly rate-limiting step of the flavonoid biosynthesis. Flavonoids are exuded by legume roots to attract rhizobia and function as a co-transcription factor in the induction of the bacterial gene expression cascade for Nod-factor formation. Silencing of genes for chalcone synthase results in impaired nodulation (Wasson et al., 2006; Abdel-Lateif et al., 2013). The increased expression of genes for chalcone synthase might represent an early reaction to the loss of nodule activity (ammonium supply) induced by the oxygen flush of the nodule interior. The objective might be to induce the formation of additional nodules in order to bridge the gap in the nitrogen supply of the higher plant that might occur in the medium term.

## Conclusion

In conclusion, the data of this report showed that acclimation of legume nodules to shifting external oxygen concentrations comprised molecular reactions that are necessary in addition to the functioning of a flexible ODB. In particular a neoformation of the enzyme nitrogenase is involved. In this context, the inner cell iron supply to the bacteroids is of pivotal importance. This highlights the importance of iron supply for legumes as a factor in the acclimation of nitrogen fixation to a changing environment. In *M. truncatula*, Medtr1g084050, a gene annotated as ‘nicotianamine synthase-like protein’ in the gene model Mt4.0v1, is of particular importance for the nodule iron turnover. Increased expression of NCR peptides might be essential to force the symbiont to increase nitrogenase *de novo* synthesis. Finally, the data showed that comprehensive, comparative transcriptome profiling could also be useful in unraveling short-term acclimation processes in plant tissue to a changing environment.

## Conflict of Interest Statement

The authors declare that the research was conducted in the absence of any commercial or financial relationships that could be construed as a potential conflict of interest.

## Footnotes

1 www.ncbi.nlm.nih.gov/tools/primer-blast

2 http://gb.sc.noble.org/cgi-bin/gb2/gbrowse/medicago_4/?name=chr1%3A37484310..37485662

## Supplementary Material

**TABLE S1 | Primer sequences used for the analysis of relative expression of *nif-* and *fix*-genes during exposure of nodules to elevated external oxygen concentrations.** **TABLE S2 | Genes expressed in nodules in a control and after 30 min exposure to 25% external oxygen concentration.** The sequenced reads were aligned to the gene model Mt4.0v1. Only ‘unique hits’ were counted. Data were normalized using the DESeq procedure (*n* = 3). A gene is considered expressed when the count number exceeds 20 in either the control, the treatment or both. 16 615 transcript units out of 62 319 annotated in the gene model met this criterion. **TABLE S3 | Transcript units with a log_2_ fold change (FC) greater than [2].** **TABLE S4 | Normalized transcript abundance of all transcript units annotated in Mt4.0v1.**
